# Ⅰ期非小细胞肺癌患者肺叶切除术后心肺相关并发症种类及其危险因素分析

**DOI:** 10.3779/j.issn.1009-3419.2016.05.06

**Published:** 2016-05-20

**Authors:** 玉田 赖, 建华 苏, 铭明 王, 坤 周, 恒 杜, 健 黄, 国卫 车

**Affiliations:** 1 610041 成都，四川大学华西医院胸外科 Department of Thoracic Surgery, West China Hospital, Sichuan University, Chengdu 610041, China; 2 610041 成都，四川大学华西医院康复科 Department of Rehabilitation, West China Hospital, Sichuan University, Chengdu 610041, China

**Keywords:** 肺肿瘤, 心肺相关并发症, 危险因素, Lung neoplasms, Postoperative cardio-pulmonary complications, Risk factors

## Abstract

**背景与目的:**

随着医学技术的进步，医学检测手段的更新，体检筛查的普及以及社会健康意识的提高，越来越多的早期肺癌能够得到及时的发现并接受手术治疗，而关于患者术后并发症发生种类和高危因素的研究很少；针对Ⅰ期非小细胞肺癌（non-small cell lung cancer, NSCLC）术后并发症及其高危因素的研究，可为此类患者术后心肺相关并发症的预防、干预提供依据，并加速患者康复。

**方法:**

回顾性分析2012年1月-2013年12月四川大学华西医院胸外科行肺叶切除的Ⅰ期NSCLC患者421例，以术后30天是否发生心肺相关并发症分为：并发症组和无并发症组。

**结果:**

最终421例患者被纳入研究，其中64例为并发症组（15.2%, 64/421），357例为非并发症组（84.8%, 357/421）。421例患者中，发生肺部感染的比例最高（8.8%, 37/421），其他主要的并发症包括肺不张（5.9%, 25/421）、中量以上胸腔积液（5.0%, 21/421），持续性肺漏气（3.6%, 15/421）等；术前合并慢性阻塞性肺部疾病（chronic obstructive pulmonary disease, COPD）（*P*=0.027），术前白细胞计数（*P* < 0.001），中性淋巴比（neutrophil-lymphocyte ratio, NLR）（*P* < 0.001），术中出血量（*P*=0.034）以及手术时间（*P*=0.007）在两组间差异有统计学意义；采用二分类Logistics回归分析后发现，术前白细胞计数（OR=1.451, 95%CI: 1.212-1.736, *P* < 0.001）、术前合并COPD（OR=0.031, 95%CI: 0.012-0.078, *P* < 0.001）是术后发生心肺相关并发症的独立危险因素。

**结论:**

术前白细胞计数以及术前合并COPD是Ⅰ期肺癌患者术后心肺相关并发症发生的独立危险因素，可能可以作为预测术后心肺相关并发症的可靠指标。

## Abstract (en)

**Background and objective:**

There are incresing lung cancer patients detected and diagnosed at the intermediate stage when the pre-malignant or early lesions are amenable to resection and cure, owing to the progress of medical technology, the renewal of detection methods, the popularity of medical screening and the improvement of social health consciousness. The aim of this study is to investigate the risk factors of the occurrence of postoperative cardio-pulmonary complications in stage Ⅰ non-small cell lung cancer (NSCLC) patients, based on routine laboratory tests, basic characteristics, and intraoperative variables in hospital.

**Methods:**

The 421 patients after lobectomy in patients with stage Ⅰ NSCLC at the West China Hospital of Sichuan University from January 2012 to December 2013 were included into the study and stratified into complication group and non-complication group, according to whether to occur postoperative cardio-pulmonary complications after lobectomy in 30 days.

**Results:**

Of them, 64 (15.2%) patients were finally identified and selected into the complication group, compared with 357 (84.8%) in non-complication group: pneumonia (8.8%, 37/421) was the primary complication, and other main complications included atelectasis (5.9%, 25/421), pleural effusion (≥middle) (5.0%, 21/421), persistent air leak (3.6%, 15/421); The operation time (*P*=0.007), amount of blood loss (*P*=0.034), preoperative chronic obstructive pulmonary disease (COPD) (*P*=0.027), white blood cell (WBC) count (*P* < 0.001), neutrophil-lymphocyte ratio (NLR) (*P* < 0.001) were significantly different between the two groups. According to the binary logistics regression analysis, preoperative COPD (OR=0.031, 95%CI: 0.012-0.078, *P* < 0.001) and WBC count (OR=1.451, 95%CI: 1.212-1.736, *P* < 0.001) were independent risk factors for postoperative cardio-pulmonary complications.

**Conclusion:**

Among an array of clinical variables in hospital, operation time, preoperative white blood cell count, preoperative COPD, may be the independent risk factors of the occurrence of postoperative cardio-pulmonary complications.

随着医学技术的进步，医学检测手段的更新，体检筛查的普及以及社会健康意识的提高，越来越多的早期肺癌能够得到及时的发现，并接受手术治疗。体检筛查技术使很多肺癌患者在并无明显症状下能够被检出，并得到及时有效的医治，而低剂量螺旋计算机断层扫描（computed tomography, CT）在肺癌高危人群体检中的广泛应用，使早期肺癌患者的检出率大大提高^[1, 2]^。外科手术被认为是早期肺癌治疗的最佳方式^[2, 3]^，尤其是近年来以胸腔镜肺叶切除术（video-assisted thoracic surgery, VATS）为代表的微创胸外科的出现，使广大早期肺癌患者获得较好的治疗^[4-9]^。心肺相关并发症肺癌术后是常见的问题，其发生延长患者术后住院时间，增加住院费用和术后死亡风险，影响术后康复及预后^[8]^。早期肺癌患者的检出率的增加，使得如何有效评估这类肺癌患者术后心肺相关并发症的风险并进行预防成为重要的临床工作之一。我们通过对单个中心2年间Ⅰ期非小细胞肺癌（non-small cell lung cancer, NSCLC）行肺叶切除术的患者的并发症进行分析，旨在发现这类患者心肺相关并发症的相关危险因素，并为有效评估肺癌患者术后心肺相关并发症的风险提供有力的证据。

## 1 对象与方法

### 1.1 研究对象

2012年1月-2013年12月四川大学华西医院胸外科住院的肺癌患者1, 097例，最终纳入患者421例。纳入标准：①术后病理诊断为Ⅰ期原发性NSCLC；②手术方式为肺叶切术+系统淋巴结清扫术；③术前无心律失常、心肌梗死等心脏病病史者；④术前未应用抗生素治疗。排除标准：①术后诊断为非Ⅰ期NSCLC或Ⅰ期小细胞肺癌；②术前有明确肺部感染史或心血管相关病史；③应用各种抗生素史，包括肺部感染常用的头孢类（如头孢咪唑、头孢硫脒等）、喹诺酮类（如莫西沙星等）等或接受长期中/中成药治疗，如雷公藤多苷、青藤碱等此类对人体免疫系统有明确影响者；④术后临床资料不完整者。

### 1.2 研究指标

主要包括术前合并疾病、术前白细胞计数、血小板计数、血红蛋白值、中性-淋巴细胞比（neutrophil-lymphocyte ratio, NLR）、白蛋白-球蛋白比（albumin/globulin ratio, A/G ratio）、尿素氮、手术时间、术中出血量、术后住院时间等。

### 1.3 手术方式

VATS手术方式应用单向式胸腔镜肺叶切除法+系统淋巴结清扫^[10, 11]^。系统淋巴结清扫左侧必须清扫第5、6、7、8、9、10组淋巴结，右侧包括第2、3、4、7、8、9、10组淋巴结。

### 1.4 心、肺相关并发症

心肺相关并发症主要包括心律失常、心力衰竭、肺部感染、中到大量胸腔积液、持续性肺漏气（>7 d）、严重皮下气肿、呼吸衰竭、成人呼吸窘迫综合征等（表 1）。心律失常主要包括房性扑动、阵发性室上性心动过速、频发性室性期前收缩；严重皮下气肿指患者同侧、对侧胸壁、头面颈部甚至背部出现大量皮下气肿；肺部感染的诊断标准如下：①痰或胸水中查到病原菌；②患者出现白细胞计数升高（开胸>15×10^9^；胸腔镜>12×10^9^^[9]^和/或体温>38 ℃，持续3日或经抗生素处理后明显下降，即可诊断肺部感染；③术后增加或调整抗生素后，症状或体征减轻或消失，白细胞计数明显下降，或者影像学显示浸润灶相应缩小，即诊断为肺部感染。

**1:** 心肺相关并发症诊断标准 Criterion of cardio-pulmonary complications

Arhythmia	Requiring pharmacological treatment or interventions by cardiologists
Pneumonia	1: Fever as seen by raised oral temperature of >38 ℃ with no focus on outside of the lungs. The highest temperature within the previous 24 h will be reported; 2: High WBC count (open >15×10^9^, VATS >12×10^9^) or persistently rises over 72 h; 3: Positive signs of infection on sputum microbiology; 4: Prolongation, upgrade or alteration of the antibiotics use; new infiltration range(s) can be found in CT/CR image(s) or shrinks after using antibiotic.
Atelectasis	1: Chest radiograph showing atelectasis/consolidation; 2: Presenting dyspnea or SpO_2_ < 90% on room air.
Pulmonary embolism	1: Pulmonary artery angiography showing embolism; 2: Dyspnea or SpO_2_ < 90%; remission after using anticoagulant.
Respiratory/heart failure or ADRS	Needing trachea cannula, Ventilator maintenance or intensive care
Bronchopleural fistula	Bronchofiberscope showing bronchopleural fistula
Aerodermectasia	1: Persisting >15 d; 2: Requiring subcutaneous incision or surgical treatment.
Hemoptysis	1: Persisting >3 d and inefficacy of pharmacological treatment; 2: Requiring surgical treatment.
Pleural effusion (≥middle)	1: Drainage time >15 d; 2: Requiring re-indwelling drainage tube.
Chylothrax	1: Raily drainage volume >500 mL (>3 d, under ambrosia condition) and requiring pharmacological treatment (like interleukin-2); 2: Requiring surgical treatment.
Air leak	1: Persisting for over 15 d; 2: Needing interventions like re-indwelling drainage tube, vacuum suction (>7 d), or surgical treatment.
WBC: white blood cell; VATS: video assisted thoracic surgery; CT: computed tomography.	

### 1.5 心肺相关并发症危险因素分析

分组后，对纳入的术前（如：术前合并疾病、术前白细胞计数、血小板计数、血红蛋白值、NLR、A/G、尿素氮等）以及术中指标（包括手术时间、术中出血量、手术方式、切除范围等）进行对比分析；统计学上存在意义的指标则经筛选后进行*Logistic*二元回归分析，得出独立危险因素。

### 1.6 统计学方法

应用SPSS 19.0（IBM Corp, Armonk, NY, USA）软件分析结果，计量资料采用均数±标准差（Mean±SD）表示，比较采用t检验；计数资料用实际例数及百分比表示，计数资料间比较采用独立样本的卡方检验或*FISH*检验。术后心肺并发症的相关风险因素进行多因素分析，多因素分析运用*Logistic*二元回归分析。*P* < 0.05为差异有统计学意义。

## 2 结果

### 2.1 基本资料

纳入的421例患者，平均年龄为（60.3±10.0）岁（47岁-78岁），其中60.6%（255/421）为男性患者，39.4%（166/421）为女性患者；吸烟患者193例（45.8%），非吸烟患者228例（54.2%）。并发症组64例（15.2%），无并发症组357例（84.8%）（表 2）。

**2:** 患者基本资料 Baseline characteristics of patients

	Complication group	Non-complication group	*P*
No. of patients	64 (15.2%)	357 (84.8%)	
Age (yr, mean±SD)	60.2±9.8	60.3±10.0	0.927
Gender			0.732
Female	24 (37.5%)	142 (39.8%)	
Male	40 (62.5%)	215 (60.2%)	
Smoking			0.469
Never	32 (50.0%)	196 (54.9%)	
Current or formal	32 (50.0%)	161 (44.1%)	
Comorbidities			
COPD	20 (31.3%)	68 (19.0%)	0.027
Hypertension	15 (23.4%)	64 (17.9%)	0.506
Diabetes	12 (18.8%)	61 (17.1%)	0.746
COPD: chronic obstructive pulmonary disease.			

### 2.2 421例Ⅰ期NSCLC术后心肺相关并发症的发生及各类发生率分析

421例患者中，发生肺部感染的比例最高（8.8%, 37/421），其他主要的并发症包括肺不张（5.9%, 25/421）、中量以上胸腔积液（5.0%, 21/421），持续性肺漏气（3.6%, 15/421）等（表 3）。

**3:** 患者术后心肺相关并发症发生情况 Details of postoperative cadio-pulmonary complications

Clinical manifestations	Cases	Rate (%)
Pneumonia	37	8.8
Persistent air leak (>7 d)	15	3.6
Atelectasis	25	5.9
Severe aerodermectasia	11	2.6
Arhythmia	7	1.7
Pleural effusion (≥Middle-plenty)	21	5.0
Hematopneumothorax or empyema	6	1.4
Respiratory failure	2	< 1.0
Heart failure	1	< 1.0
ARDS	2	< 1.0
Chylothrax	2	< 1.0
Bronchopleural fistula	1	< 1.0
Acute hypercapnia	2	< 1.0
Prolonged mechanical ventilation (>48 h)	3	< 1.0
Pneumonedema	3	< 1.0
ARDS: adult respiratory distress syndrome.		

### 2.3 术后并发症发生的高危因素

并发症组患者术前合并COPD[31.3% (20/64) *vs* 19.0% (68/357), *P*=0.027]、术前白细胞计数[(8.1±2.7)×10^9^/mL *vs* (6.1±2.0)×10^9^/mL, *P* < 0.001]，NLR比值[(4.1±3.2) *vs* (2.6±1.7), *P* < 0.001]，术中手术时间[(138.1±47.1) min *vs* (118.5±53.9) min, *P*=0.007），术中出血量[(130.5±73.7) mL *vs* (113.2±79.3) mL, *P*=0.034]均显著高于无并发症组（表 4）。采用*Logistic*回归分析发现，术前白细胞计数（OR=1.451, 95%CI: 1.212-1.736, *P* < 0.001），术前合并COPD（OR=0.031, 95%CI: 0.012-0.078, *P* < 0.001）是术后发生心肺相关并发症的独立危险因素（表 5）。

**4:** 两组患者临床特征比较 Comparison of clinical features between the two groups

	Complication group	Non-complication group	*P*
No. of patients	64 (15.2%)	357 (84.8%)	
Preoperative variables			
WBC (×10^9^/L)	8.1±2.7	6.1±2.0	< 0.001
HB (g/L)	137.6±15.1	136.7±15.4	0.678
PLT (×10^9^/L)	176.4±63.1	174.6±53.7	0.810
NLR	4.1±3.2	2.6±1.7	< 0.001
A/G ratio	1.7±0.4	1.7±0.8	0.948
Urea nitrogen (mmol/L)	6.6±4.6	6.0±5.1	0.453
Intraoperative variables			
Operation time (min)	138.1±47.1	118.5±53.9	0.007
Amount of blood loss (mL)	130.5±73.7	113.2±79.3	0.034
Surgical approach			0.552
VATS	49 (76.6%)	285 (79.8%)	
Open	15 (23.4%)	72 (20.2%)	
Resection location			0.479
RUL	21 (32.8%)	117 (32.8%)	
RLL	11 (17.2%)	67 (18.8%)	
RML	3 (4.7%)	26 (7.3%)	
LUL	19 (29.7%)	90 (25.2%)	
LLL	10 (15.6%)	57 (16.0%)	
Pleural invasion			
Yes	33 (51.6%)	185 (51.8%)	0.618
No	31 (48.4%)	172 (48.2%)	
Antibiotics use			
Postoperative use time (d)	6.8±2.9	3.9±2.5	< 0.001
Categories of antibiotics			
Second-generation cephalosporins	43 (67.2%)	264 (73.9%)	0.467
Third-generation or senior	21 (32.8%)	93 (26.1%)	
Duration of postoperative hospital stay (d)	7.9±3.1	5.2±2.7	< 0.001
WBC: white blood cell; PLT: platelet; HB: hemoglobin; NLR: neutral-lymph ratio; VATS: video assisted thoracic surgery; RUL: right upper of lung; RLL: right lower of lung; RML: right middle of lung; LUL: left upper of lung; LLL: left lower of lung.			

**5:** 独立危险因素回归分析 *Logistic* regression analysis

Features	Odds ratio	95% confidence interval		*P*
		Lower bound	Upper bound	
Operation time	1.004	0.998	1.010	0.215
Amount of blood loss	1.002	0.999	1.004	0.142
COPD	0.031	0.012	0.078	< 0.001
WBC	1.451	1.212	1.736	< 0.001
NLR	0.938	0.780	1.129	0.500
Constant	0.152			0.007

### 2.4 两组患者抗生素应用及住院时间分析

相比于非并发症组，抗生素应用时间[(6.8±2.9) d *vs* (3.9±2.5) d, *P* < 0.001]和术后住院时间[(7.9±3.1) d *vs* (5.2±2.7) d, *P* < 0.001]在并发症组显著长于无并发症组（表 4）。

## 3 讨论

有效的评估、预测肺癌手术患者术后心肺相关并发症的发生风险，有助于临床医务工作者合理展开防治工作，指导制定相应治疗方案，尤其是个体化的抗生素使用。本研究目的在分析Ⅰ期NSCLC患者发生术后心肺相关并发症的相关危险因素，为进一步研究有效评估、预测此类肺癌患者术后心肺相关并发症发生提供证据和基础。

抗生素在肺癌手术患者，尤其是术后感染风险高的患者的围术期的使用被普遍接受和认可。而目前仍未有针对胸外科手术的统一、规范的临床指南用来指导合理临床用药。目前多数医院仍常规使用一代或二代头孢进行术后感染预防，尽管有研究^[12, 13]^认为使用一代或二代头孢能有有效降低术后伤口感染，但并不能明显降低术后肺部感染的发生率。本次研究结果发现肺部感染仍然是Ⅰ期NSCLC患者术后最常见的并发症，其他主要的并发症包括肺不张、持续性肺漏气、中量以上胸腔积液等等。较高的肺部感染发生率，不可避免地增加了患者术后护理的抗生素使用频率，延长了术后住院时间；同时在Ⅰ期NSCLC患者中，并发症组的患者术后抗生素的使用时间、术后平均住院时间更长，使用三代或更高级别抗生素的比例更高（尽管差异并没有统计学意义），增加患者住院费用的同时更影响患者术后的快速康复。因此有效筛选、评估心肺相关并发症的高危因素，制定合理的感染预防、治疗措施降低其发生率，对减少患者的术后康复时间，加速其快速康复具有重要作用。

COPD与肺功能的降低密切相关，其与术后并发症的发生的相关性已经被大量研究证实^[14, 15]^。肺癌合并COPD的手术患者，无论在手术风险上还是在术后康复上（如术后心肺相关并发症发生率、肺功能恢复情况等），都存在较大的挑战。研究结果也显示术前合并COPD是术后发生心肺并发症的独立危险因素。术前合并COPD的患者，可能是术后心肺相关并发症发生的高危人群，进一步的研究可把是否患有COPD作为其风险评估的重要参考指标。

有研究^[16, 17]^指出，术前肺部感染或肺炎病史的手术患者是术后发生肺部感染的高危人群。手术前合并肺部感染或肺炎病史极有可能增加术后肺部感染的发生风险。术前合并肺部感染或肺炎病史，经治疗后可能术前白细胞计数值仍然维持在一个较高的水平，同时白细胞计数水平较高可能也提示患者体内较高水平的炎症状态，潜在地增加术后肺部感染的发生率。本研究结果显示术前白细胞计数是术后心肺并发症发生的独立危险因素，提示其可作为评估发生风险的一项有效指标，而这一结果在既往的研究中罕被提及，尽管其有效性以及可推广性有待研究进一步证明。同时，手术时间过长，麻醉风险和患者术后感染风险相应增加。结果分析也表明，手术时间是术后心肺相关并发症的危险因素（非独立危险因素）。

本研究结果显示年龄并不是一个术后心肺相关并发症的独立危险因素，但是有研究^[16]^已经证明其对手术患者术后心肺并发症的重要影响，其原因可能为早期肺癌患者普遍年龄较轻，年龄因素在术后心肺相关并发症的影响并非在主导地位等等。同样，吸烟和患者的肺功能、COPD的发生，心血管疾病的发生密切相关，可能也是肺癌手术患者术后出现心肺相关并发症的重要相关因素，尽管结果并没有支持这一观点。此外，高血压与心血管疾病关系密切，其与心肺相关并发症的联系亦有待进一步的探讨。

作为一项单中心的回顾性分析，本研究也存在一定的局限性。我们依据纳入/排除标准排除了部分患者（纳入的患者仅为2012年-2013年四川大学华西医院胸外科Ⅰ期NSCLC手术患者），不可避免地降低了研究结果的推广性和普遍可行性；针对的患者人群仅为Ⅰ期的原发性NSCLC患者，需要进一步的研究来证明其结论的有效性能否用于中晚期的肺癌患者，而这也是实验的下一步计划；由于目前尚无统一的心肺相关并发症的诊断标准，造成研究结果的可比性、可推广性存在局限；由于纳入分析的指标有限，更多的指标需要在未来的研究中补充、纳入进来，进一步完善研究分析等。

通过本次研究，我们发现术前白细胞计数以及术前合并COPD是Ⅰ期原发性NSCLC手术患者术后心肺相关并发症发生的独立危险因素，可以作为心肺相关并发症发生的风险评估的有效指标。
